# Effect of angiotensin II type 1 receptor blockade on kidney ischemia/reperfusion; a gender-related difference

**DOI:** 10.15171/jrip.2016.29

**Published:** 2016-07-28

**Authors:** Fatemeh Moslemi, Pegah Taheri, Mahdis Azimipoor, Sina Ramtin, Mostafa Hashemianfar, Ali Momeni- Ashjerdi, Fatemeh Eshraghi-Jazi, Ardeshir Talebi, Hamid Nasri, Mehdi Nematbakhsh

**Affiliations:** ^1^Water & Electrolytes Research Center, Isfahan University of Medical Sciences, Isfahan, Iran; ^2^Department of Physiology, Isfahan University of Medical Sciences, Isfahan, Iran; ^3^IsfahanMN Institute of Basic & Applied Sciences Research, Isfahan Iran

**Keywords:** Ischemia reperfusion injury, Losartan, Gender, Acute kidney injury

## Abstract

**Background:** Renal ischemia/reperfusion (I/R) injury may be related to activity of reninangiotensin system (RAS), which is gender-related. In this study, it was attempted to compare the effect of angiotensin II (Ang II) receptor type 1 (AT1R) blockade; losartan in I/R injury in male and female rats.

**Materials and Methods:** Male and female Wistar rats were assigned as sham surgery, control I/R groups treated with vehicle, and case I/R groups treated with losartan (30 mg/kg). Vehicle and losartan were given 2 hours before bilateral kidney ischemia induced by clamping renal arteries for 45 minutes followed by 24 hours of renal reperfusion.

**Results:** The I/R injury significantly increased the serum levels of blood urea nitrogen (BUN) and creatinine (Cr), and kidney tissue damage score in both genders. However, losartan decreased these values in female rats significantly (*P* < 0.05). This was not observed in male rats.

**Conclusion:** Losartan protects the kidney from I/R injury in female but not in male rats possibly because of gender-related difference of RAS.

**Implication for health policy/practice/research/medical education:**

Losartan acts differently in ischemia/reperfusion injury in male and female.

## Introduction

Renal ischemia/reperfusion (I/R) injury is the most common cause of acute kidney injury (AKI), which is associated with increased morbidity and mortality rates in clinic. It occurs in hypovolemic conditions, septic shock, renal transplantation, or cardiovascular surgery (1). Generation of reactive oxygen species (ROS) highly increases during ischemia and reperfusion, and the overproduction of ROS promotes cell membrane peroxidation and inhibits antioxidant system and causes mitochondrial dysfunction (2). It is known that ischemia in extensive level leads to necrosis of proximal and outer medulla parts (3). Renin-angiotensin system (RAS) plays a predominant role in blood pressure regulation in both genders. The end product of RAS, angiotensin II (Ang II), binds to two main subtypes of receptors; namely angiotensin II type 1 and 2 receptors (AT1R and AT2R). This system has a significant role in I/R process, and it is reported that losartan, an AT1R blocker, inhibits elevation of serum creatinine (Cr) level induced by I/R injury (4). However, the AT1R expression is higher in males while females have higher AT2R expression levels; therefore, kidney circulation may be different between the genders (5), and male hormone; testosterone may contribute to development of I/R injury in mice (6,7).

Losartan is usually prescribed for hypertension treatment. It is also known that the RAS activity may be gender-related (5,8). Therefore, we hypothesized that AT1R blockade may provide a gender-related effect in I/R injury model in rats.

## Materials and Methods

Thirty-four age-matched male and female Wistar rats were used in this study (18 male and 16 female). The sham groups (group 1: male, n=6, and group 2: female, n=6) were subjected to sham surgery. Control groups (group 3: male, n=5, and group 4: female, n=5) received saline as vehicle two hours before induction of ischemia. The case groups (group 5: male, n=7, and group 6: female, n=5) were treated the same as control groups except they received losartan (30 mg/kg) instead of vehicle. Bilateral kidney ischemia was induced by clamping renal arteries for 45 minutes. Then, the clamps were removed to induce renal reperfusion. Twenty-four hours post reperfusion, the animal was anesthetized again, blood samples were obtained via heart puncture, and after sacrificing the animals, the left kidney was removed for staining and pathology investigation. Right kidney was homogenized and centrifuged to obtain supernatant for the measurement.

The serum levels of Cr and blood urea nitrogen (BUN) were measured by quantitative diagnostic kits (Pars Azmoon, Tehran, Iran). Serum and tissue levels of nitrite were measured by an assay kit that involves the Griess reaction. Malondialdehyde (MDA) levels in serum and tissue were also measured manually. The left kidney was kept in 10% formalin solution and embedded in paraffin. The samples were stained by hematoxylin and eosin (H&E), and the tissue damage was determined by two pathologists who were totally blind to the study protocol. Accordingly, the kidney tissue damage was determined based on tubular membrane loss, brush borders and tubular dilation and simplification, and tubular cell swelling and necrosis. Finally, injury proportion (%) was reported.

### Ethical issues

The research followed the tenets of the Declaration of Helsinki. The research was approved by ethical committee of Isfahan University of Medical Sciences. Prior to the experiment, the protocols were confirmed to be in accordance with the Guidelines of Animal Ethics Committee of Isfahan University of Medical Sciences.

### Statistical analysis

The data are expressed as mean ± SEM. The groups were compared in terms of the serum levels of BUN, Cr, nitrite, and MDA; and tissue nitrite and MDA levels using the one-way analysis of variance (ANOVA) followed by the least significant difference (LSD) test. The kidney tissue damage score (KTDS) was analyzed using the Kruskal-Wallis and Mann-Whitney U tests. The *P* value less than 0.05 was considered significant.

## Results

### Effects of losartan on serum levels of BUN and Cr, kidney weight and KTDS

**Figure 1:**
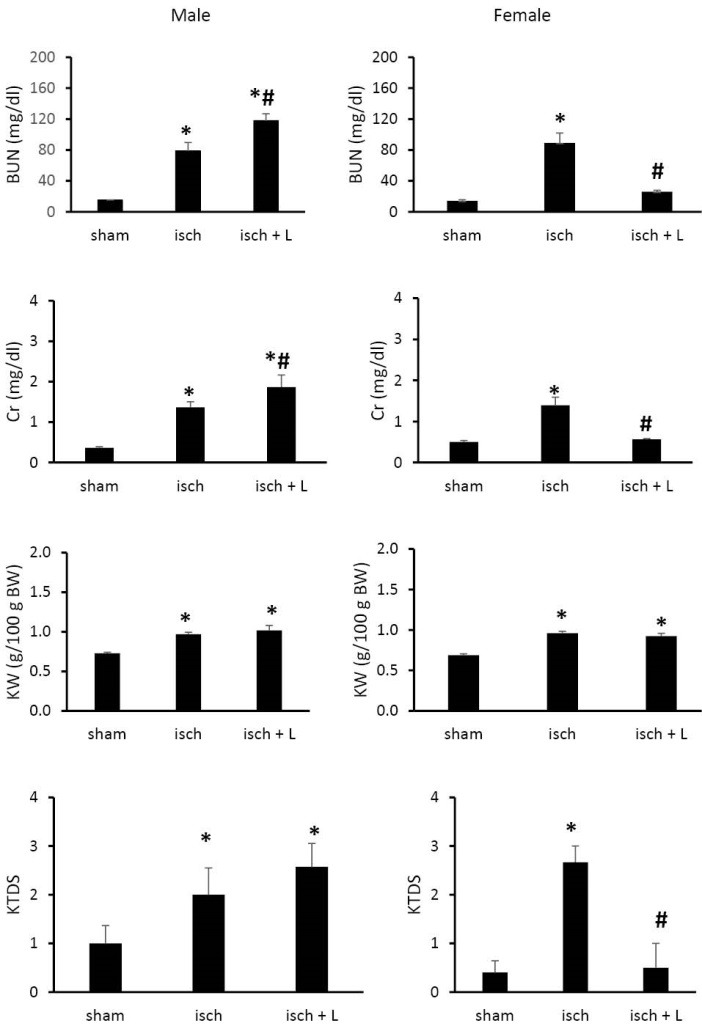
Serum levels of BUN and Cr, KW and KTDS in male and female rats as sham-operated (sham), ischemia/reperfusion (I/R) (isch), and I/R treated with losartan (isch + L) groups. * and # indicate significant difference (*P*<0.05) from the sham and isch groups, respectively.

The I/R injury significantly increased the serum levels of BUN and Cr in both genders when compared with the sham group. However, this increase was higher by losartan in males. Such observation was not seen in females, and losartan attenuated the induced increase of serum levels of BUN and Cr by I/R injury. Such findings were in line with the data obtained for kidney weight (KW) and KTDS (Figure 1).

### Effects of losartan on serum and kidney levels of nitrite and MDA and bodyweight

The groups; neither male nor female groups, demonstrated no significant changes in serum and kidney levels of nitrite and MDA, and in bodyweight (BW) change (Table 1).

**Table 1:** Bodyweight change (∆BW), serum levels of nitrite and MDA (SN, SMDA), and kidney nitrite and MDA (KN, KMDA) in male and female rats as sham-operated (sham), I/R (isch), and I/R treated with losartan (isch + losartan) groups

**Group**	**∆BW (g)**		**SN (µmole/l)**		**SMDA (µmole/l)**		**KN (µmole/g tissue)**		**KMDA (nmole/g tissue)**	
	**Male**	**Female**	**Male**	**Female**	**Male**	**Female**	**Male**	**Female**	**Male**	**Female**
Sham	6.8 ± 2.2	7.3 ± 0.6	7.3 ± 1.7	6.4 ± 1.1	4.6 ± 0.4	4.1 ± 0.3	0.24 ± 0.01	0.27 ± 0.02	8.9 ± 1.5	6.7 ± 1.4
I/R	11.4 ± 2.0	1.8 ± 4.3	6.7 ± 0.7	6.9 ± 1.2	4.1 ± 0.2	3.7 ± 0.4	0.21 ± 0.03	0.23 ± 0.06	4.4 ± 0.4^a^	5.8 ± 1.0
I/R+ losartan	7.7 ± 1.1	5.0 ± 0.7	7.6 ± 1.4	8.0 ± 3.4	4.0 ± 0.2	4.3 ± 0.4	0.18 ± 0.01^a^	0.24 ± 0.01	7.2 ± 0.7	6.5 ± 0.8
*P* (ANOVA)	0.22	0.28	0.9	0.85	0.34	0.77	0.05	0.62	0.03	0.84

^a^Significant difference from sham group (*P*<0.05

## Discussion

In this study, we attempted to investigate the effect of losartan as AT1R blocker on renal I/R in male and female rats. We demonstrated that pretreatment with losartan accelerated kidney injury in male but not in female rats. Ischemia occurs in arterial occlusion, organ transplantation, and shock and leads to cell and organ injury. Following kidney ischemia, when reperfusion occurs, it lead to further injury in tissue and development of acute renal failure (ARF) (9). In the present study, we observed ARF induced by renal I/R injury in male and female rats, which was characterized by increase in BUN and Cr serum levels, KW, and KTDS (10).

The RAS has a role in regulation of blood pressure in both genders and exert pivotal effect in progression and enhancement of renal injury (11). Ang II exerts its effect via two receptor subtypes; AT1R and AT2R (12). Studies have indicated that AT2 receptor plays contrary roles in regulation of blood pressure in males, which can be seen as a vasodilatory response (13).

It has been demonstrated that females have higher renal AT2:AT1 receptor ratio than males. It provides evidence for vasoconstrictor/vasodilator balance of RAS in males and females (14), and the contribution of this system in development of cardiovascular diseases can be gender-related (15).

Ang II is one of the important vasoactive agents involves the ROS production during ARF (16) via overexpression and action of NADPH (17). High level of Ang II during reperfusion period causes deleterious effect in kidney tissue such as necrosis and apoptosis (2) and reduction in mRNA level of AT1R (4). Accordingly, losartan plays an important role in systemic hemodynamic in ARF (4). In addition, it is well known that males are more sensitive to I/R-induced kidney injury than females; possibly due to sex hormone. Testosterone inhibits nitric oxide synthase activity and result in greater inflammatory response (6), and worsens renal injury in kidney diseases (18). In this regard, the risk of cardiovascular disease in males and menopause women is related to the protective effect of estrogen (19).

Females are protected against I/R injury because sexual hormones affect expression of cytokinin and vascular regulatory factors that are released after ischemia injury (20). Some evidence showed that the reason for these differences is the effect of gender on RAS; as sexual hormones and gender affect Ang II and AT1R . In conclusion, as AT2R receptor expression in females is greater than that in males, it can be considered that inhibition of AT1R by losartan alter renal blood flow differently in males and females (21), and it increases stimulation of AT2R receptor in the kidney, while AT2 has a vasodilatory effect caused to elevate perfusion and GFR in kidney. Therefore, it can improve IRI and renal hemodynamics in female rats. It is also indicated that estrogen upregulate AT2R in females (14,22) and protect females against Ang II-induced hypertension (15,23).

## Authors’ contribution

FM was involved in animal experiment, data analysis and preparing the primary draft. PT, MA, SR, MH and AMA contributed equally to the manuscript by handling the experimental research and preparing the primary draft. FEJ helped in collection and analyzing the data. AT and HN commented, gave their advice and helped during the research for pathology findings. MN designed and supervised the research and data analysis, and prepared the final manuscript. All authors read and approved the final manuscript.

## Conflicts of interest

The authors declare that they have no conflicting interest.

## Ethical considerations

Ethical issues (including plagiarism, data fabrication, double publication) have been completely observed by authors.

## Funding/Support

This research was supported by Isfahan University of Medical Sciences (Grant# 293364).
